# Genome-scale metabolic network model of *Eriocheir sinensis i*crab4665 and nutritional requirement analysis

**DOI:** 10.1186/s12864-022-08698-z

**Published:** 2022-06-28

**Authors:** Jingjing Li, Yifei Gou, Jiarui Yang, Lingxuan Zhao, Bin Wang, Tong Hao, Jinsheng Sun

**Affiliations:** 1 Tianjin Fisheries Research Institute, Tianjin, China; 2 Tianjin Key Laboratory of Animal and Plant Resistance, College of Life Sciences, Tianjin Normal University, Tianjin, China

**Keywords:** Genome-scale metabolic network_1_, network analysis_2_, biomass_3_, nutrient requirement_4_, Eriocheir sinensis_5_

## Abstract

**Background:**

Genome-scale metabolic network models (GEMs) provide an efficient platform for the comprehensive analysis the physical and biochemical functions of organisms due to their systematic perspective on the study of metabolic processes. *Eriocheir sinensis* is an important economic species cultivated on a large scale because it is delicious and nutritious and has a high economic value. Feed improvement is one of the important methods to improve the yield of *E. sinensis* and control water pollution caused by the inadequate absorption of feed.

**Results:**

In this study, a GEM of *E. sinensis*, *i*crab4665, was reconstructed based on the transcriptome sequencing, combined with KEGG database, literature and experimental data. The *i*crab4665 comprised 4665 unigenes, 2060 reactions and 1891 metabolites, which were distributed in 12 metabolic subsystems and 113 metabolic pathways. The model was used to predict the optimal nutrient requirements of *E. sinensis* in feed, and suggestions for feed improvement were put forward based on the simulation results. The simulation results showed that arginine, methionine, isoleucine and phenylalanine had more active metabolism in *E. sinensis*. It was suggested that the amount of these essential amino acids should be proportionally higher than that of other amino acids in the feed to ensure the amino acid metabolism of *E. sinensis*. On the basis of the simulation results, we further suggested increasing the amount of linoleic acid, EPA and DHA in the feed to ensure the intake of essential fatty acids for the growth of *E. sinensis* and promote the accumulation of cell substances. In addition, the amounts of zinc and selenium in the feed were also suggested to be properly increased to ensure the basic metabolism and growth demand of *E. sinensis*.

**Conclusion:**

The largest GEM of *E. sinensis* was reconstructed and suggestions were provide for the improvement of feed contents based on the model simulation. This study promoted the exploration of feed optimization for aquatic crustaceans from *in vivo* and *in silico.* The results provided guidance for improving the feed proportion for *E. sinensis*, which is of great significance to improve its yield and economic value.

**Supplementary Information:**

The online version contains supplementary material available at 10.1186/s12864-022-08698-z.

## Background

The demand for aquatic products has grown with the development of the global economy and the general improvement in living standards As an essential food and economic source, half of the global consumption of aquatic products comes from aquaculture. To meet the growing demand, the scale and output of aquaculture have to be increased to provide safe and healthy aquatic products. *Eriocheir sinensis*, also known as Chinese mitten crab and river crab, is an essential aquatic product because of its delicious and nutritious flesh. Every 100 g of edible crab contains about 14% protein, 5.9% fat, 7% carbohydrate, and various vitamins, chitin, niacin, trace elements and other nutrients, among which chitin account for 25-30% of the dry weight of crab shell [1]. Its nutritional composition is higher than that of most fish, which determines its high nutritional and economic values [2]. In the artificial culture of *E. sinensis*, artificial feed is usually used due to its easy management, low price and low pollution to the environment. Therefore, the types and proportions of different nutrients in the artificial feed need to be optimized for the sustainable development of the *E. sinensis* culture industry. In recent years, many researchers have tried to reveal the demand for various nutrients in the growth process of *E. sinensis* through the trial and error experiments based on nutritive physiology [3]. This helped find a more perfect and reasonable feed composition to improve the efficiency of *E. sinensis* breeding. For example, Sun Jinhui *et al.* found that the *E. sinensis* had obvious growth advantages when fed with formula feed and a natural diet containing 38% and 35% protein, respectively [4]. Sunxinjin *et al.* found that the proper addition of vitamin A in the feed promoted the growth of juvenile crab and improved feed efficiency, but the excessive addition (>160,000 IU/kg feed) inhibited the growth to a certain extent [5]. In addition, vitamin E effectively improved the anti-stress ability and immunity of crabs. For diseased crabs, an appropriate amount of vitamin E supplementation can not only reduced the mortality of shrimps and crabs, but also promoted their early recovery [6]. However, due to the complexity of the feed composition, finding the real optimal feed proportion based on the trial-and-error method is a difficult task.

With the continuous improvement of genome sequencing technology, it has become a research hotspot to study the metabolic process of organisms from a systematic perspective using omics Big Data, which promotes the rapid development of a genome-scale metabolic network model (GEM). A large number of high-throughput omics data and annotation information provide a broad database for the developing GEM [7]. The reconstruction of a GEM starts from omics sequencing data, combined with genome annotation, metabolism related database and experimental data, to reconstruct the metabolic network by intergrating gene-protein-reaction. It used in quantitative studies on the metabolic process of organisms from the perspective of systematics [8–10]. GEMs have been applied in analysising and predcting of gene expression and function, discovery of drug targets, guidance of metabolic engineering and aquaculture productions [11–13]. It has also been successfully applied to predict the nutritional requirements of several microorganisms, such as *Escherichia coli* and *Bacillus subtills* [14, 15].

In this study, a whole body GEM for growing juvenile *E. sinensis* was reconstructed based on the transcriptome sequencing data for five mixed organs and the KEGG database. The biomass equation was constructed based on the nutritional component detection of *E. sinensis* hepatopancreas, which gave GEM the ability to synthesize biomass. With flux balance analysis, the growth and the optimal nutritional requirement of *E. sinensis* were simulated and suggestions on feed improvement were proposed. This study made the analysis of nutritional requirement for *E. sinensis* take the first step from the traditional *in vivo* experiment to the *in silico* experiment. It also provided a theoretical basis for guiding the breeding industry and promoted the development of crab production.

## Methods

### Transcriptome sequencing

The healthy juvenile Chinese mitten crabs were taken from Tianjin Xieyuan aquaculture Co., Ltd. The average body length, body width and weight of crabs were 24.28 ± 1.54 mm, 26.97 ± 1.65 mm and 8.41 ± 1.47 g, respectively. The crabs were cultured in a plastic incubator (70 × 40 × 50 cm) at 20 ± 1°C with natural light. The crabs were fed compound feed twice a day. The daily feeding amount was about 5% of the body weight. The siphon method was used to clean up the residual bait and inject new water. The daily water change is more than 50%. As all metabolic activities in the non-molting stage of juvenile crab are aimed to prepare for molting. Therefore, in addition to the hepatopancreas, which is the metabolic and nutrient storage tissue, four other tissues which are closely related to molting, including gill, muscle, thoracic ganglion and eyestalk, were also selected as the sample for sequencing. While collecting the samples, the crabs were put on the ice plate for low-temperature anesthesia. The five tissues of crabs were quickly collected and put in liquid nitrogen immediately, and then, preserved in a −80°C refrigerator for preservation. Each sample contains the mixture of five tissues in a crab. All experimental procedures were conducted according to institutional guidelines for the care and use of laboratory animals in Tianjin Normal University and conformed to the National laboratory animal management regulations (Publication No. 2, 1988) approved by the National Science and Technology Commission.

Total RNA from mixed *E. sinensis* tissues was sequenced using the Illumina high-throughput sequencing technology by Novogene Inc. The standard procedure is the same with that in our previous work [16] (Supplementary file 1). The sequenced unigenes were subsequently aligned against the NR and Nt databases using BLAST searching with an E-value < 1*E-5 and the PFAM database using the HUMMER package with an E-value < 0.01. The GO annotation of unigenes was obtained using the Blast2GO program. The KO and pathway annotations were obtained by KAAS (KEGG Automatic Annotation Server). The RNA-seq data have been submitted to the Gene Expression Omnibus (GEO) database, and the accession number is GSE182818.

### Preliminary reconstruction of GEM

**Fig. 1:**
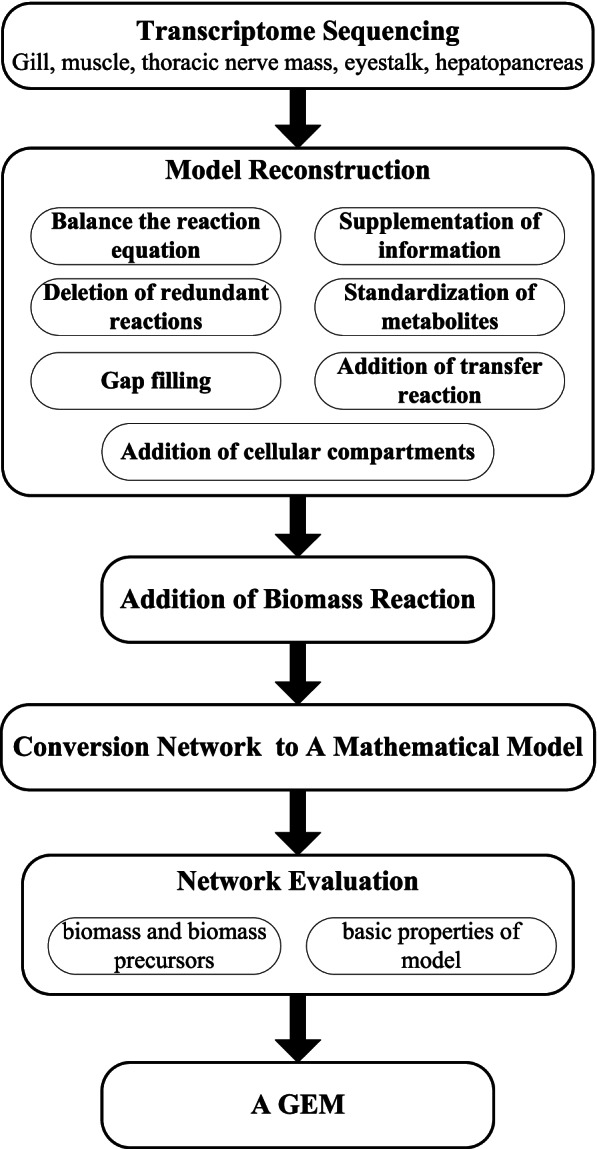
Workflow of GEM reconstruction

The reconstruction of the GEM followed the standard protocol proposed by Thiele and Palsson [17]. The reconstruction process comprised of preliminary reconstruction, refinement of GEM, addition of biomass reaction, conversion of the network into a mathematical model and network evaluation. The workflow of GEM reconstruction is shown in Fig. 1.

In the preliminary reconstruction, the related information of metabolic reactions was downloaded from the KEGG database [18], including the corresponding relationship between reaction and enzyme, pathway, subsystem, and KO. Using KO ID as a bridge, the reaction information containing KO ID in the KEGG database was matched with unigenes containing KO ID in transcriptome data, and the preliminary reconstructed GEM of *E. sinensis* obtained, which was composed of unigenes, enzymes, reactions, main reaction equations, pathways, and subsystems. Visual Basic for Applications (VBA) was used for the data assembly and following network refinement.

### Refinement of GEM

The preliminary reconstructed GEM were refined in the following aspects: (1) network balance of the reaction equactions were firstly checked and balanced with the principle of element and charge conservation; (2) the lacking information about main reactions, pathways and subsystem of some reactions were supplemented; (3) the redundant reactions such as step reaction, general reaction, incomplete reaction and macromolecular reaction were further processed to simplify the network and ensure the reasonable distribution of metabolic flux; (4) the metabolites with unclear chirality were standarlized; (5) gaps in the network were filled in the pathway scale and global scale with all the reactions in the KEGG database used for gap filling; (6) transfer reactions were added to the GEM according to the characteristics of species to fit the actual situation of biological metabolism and simulate the metabolic process more accurately; (7) the reactions in GEM were divided into two compartments: cytosol and extracellular. The detail of the refinement procedures were supplied in Supplementary file 1.

### Addition of biomass reaction

Biomass reaction reflects the growth of cells or organisms, which usually represents the composition of cells. The biomass reaction of a species is expressed by linear equation according to the proportion of all the components in the cell [19]. The hepatopancreas of *E. sinensis* is responsible for the metabolism and storage of nutrients. It is closely related to the growth and reproduction of crustaceans. Therefore, the hepatopancreas of *E. sinensis* was used for detecting of nutritional components. The hepatopancreas sample used for nutritional component detection was from the same crabs used for transcriptome sequencing. The biomass constituents in the sample were tested experimentally by the Beijing Institute of Nutritional Resources using the standard method specified in “GB28050-2011 national food safety standard general principles for nutrition labeling of prepackaged food” [20]. The contents of 20 amino acids, 22 fatty acids, 9 trace elements and 2 saccharides in the sample were tested. The biomass reaction equation of *E. sinensis* was constructed based on the nutrient composition of hepatopancreas. The unit of detection results was converted into mmol ⋅ gDW^-1^ to obtain the coefficient of biomass components. DW in the reaction flux unit represented the dry weight of cells. The proportion of each substance in the DW of cells was obtained by subtracting the weight of water from the total amount of each nutrient.

### Conversion of the network into a mathematical model

The GEM of *E. sinensis* was transformed into a mathematical model for the following simulation. MATLAB 2018b software from Mathworks (https://matlab.mathworks.com/) and COBRA toolbox v.3.0 [21] were used for model transformation and subsequent simulation. Before the simulation, the flux constraints of reactions should be limited. The flux of reversible reactions was set to be (-1000, 1000) mmol ⋅ gDW^-1^ ⋅ h^-1^, and that for irreversible reactions is (0, 1000) mmol ⋅ gDW^-1^ ⋅ h^-1^. For the nutrients that could be synthesized by the model, the flux of their transport and exchange reactions was set to be (0, 1000) mmol ⋅ gDW^-1^ ⋅ h^-1^. The flux of the transport and exchange reactions for the nutrients that needed to be absorbed from the feeds or environments was set to be (-5, 1000) mmol ⋅ gDW^-1^ ⋅ h^-1^, except for trace elements set as (-1, 1000) mmol ⋅ gDW^-1^ ⋅ h^-1^.

### Network evaluation

#### Synthesis of biomass and biomass precursors

After the refinement of the network, the GEM needed to be further evaluated for the accurate simulation of the metabolic process for some important precursors and synthesis of biomass. In this study, the synthesis of all the precursors of the biomass reaction was detected. As the trace elements and saccharides were all obtained from the feed, the precursors needing evaluation were actually all the amino acids and fatty acids. Ten essential amino acids (lysine, arginine, methionine, tryptophan, phenylalanine, leucine, histidine, valine, threonine, and isoleucine) [22] and 10 nonessential amino acids (alanine, asparagine, aspartic acid, cysteine, glutamine, glutamic acid, glycine, proline, serine and tyrosine) in *E. sinensis* were checked first. Then, the synthesis of all the fatty acids was checked, especially for 5 essential fatty acids, including linoleic acid (18:2n-6), linolenic acid (18n: 3n-3), eicosapentaenoic acid (20:5n-3, EPA), docosahexaenoic acid (22:6n-3, DHA), and arachidonic acid (20:4n-6, ARA) [23, 24]. Flux balance analysis (FBA) was used for the investigation, which is a constraint-based simulation optimization method commonly used in GEM simulation [25, 26]. The FBA assumes that the system is in a quasi steady state. Under this assumption, certain constraints and objective functions were set, and the optimal objective function value was calculated to study the flux distribution in cells under corresponding conditions. The objective function could be set according to the purpose of the study. The exchange reaction of each precursor was set to be the objective function, respectively. If the analog value for a certain precursor was 0, it reflected some defect existing in the metabolic route for this substance in the network. Then, taking the precursor as the starting point, its synthesis pathway was backtracked to see where the breakpoint appeared. After finding out the breakpoint, gap-filling reactions were added to complete the synthesis pathway. Subsequently, the synthesis of this precursor was simulated again. If it still could not be synthesized, the correction process was repeated until the precursor could be synthesized successfully. Similarly, in the investigation of biomass synthesis, the biomass reaction was set to be the objective function and its maximal flux was evaluated by the same method [27].

#### Testing basic properties of the model

For evaluating the quality of the model, the following 10 basic properties of *i*crab4665 were tested according to the Cobratoolbox tutorial (https://opencobra.github.io/cobratoolbox/stable/tutorials/tutorialModelSanityChecks.html): (1) leak test 1: whether the closed model could produce any exchanged metabolite from nothing; (2) leak test 2: if something leaked when demand reactions for each metabolite in the model were added; (3) if the model produced energy from water; (4) if the model produced matter when the ATP demand was reversed; (5) if the model had flux through the h[c] demand; (6) if the model produced too much atp demand from glucose; (7) if duplicated reactions occurred ; (8) if empty columns were present; (9) if demand reactions had a lb >= 0; and (10) whether single-gene deletion ran.

#### Analysis of optimal nutrition requirement

In the simulation of optimal nutrition requirement, the upper and lower limits of the biomass reaction were set as 1 gDW ⋅ h^-1^. The biomass reaction was set to be the objective function. The FBA was used to simulate the flux distribution in the metabolic network for 1 gDW biomass synthesis. The flux of the exchange reaction for each nutrient was the optimal nutrient absorption required for the cell to accumulate 1 g of dry weight cell.

### Specific growth rate

The growth rate of *E. sinensis* was evaluated as a specific growth rate (SGR) described in Ref. [28].

$$SGR/\%\cdot {\mathrm{d}}^{-1}=\left[\frac{\mathit{\ln}\left({m}_2\right)-\ln \left({m}_1\right)}{t_2-{t}_1}\right]\ast 100\%$$

where *m*_1_ and *m*_2_ represent the initial and final weight, respectively. *t*_1_ and *t*_2_ represent the initial and final time, respectively.

During the breeding, the actual feed for juvenile crab should be 3% - 5% weight/day and the crab we used in this study is averaged 8.41 g. Therefore, the feed for a crab per day should be 0.2523 (8.41 × 3%) to 0.4205 (8.41 × 5%) g. Assuming that all the feed can be absorbed and used for growth, if the biomass synthesis rate is 1.0 gDW ⋅ d^−1^, the feed requirement rate is 1.0 g/gDW^−1^d^−1^. So the nutrients absorbing rate used in the simulation was the rate for 1 g feed per day. Therefore, if the simulated biomass synthesis rate was *x* gDW ⋅ d^-1^, then the biomass synthesis rate for 1-day amount feed should be 0.2523 *x* to 0.4205 *x* gDW ⋅ d^-1^. Considering this was the dry weight excluding 51.6% water, the fresh biomass synthesis rate should be 0.2523 *x* /(1-51.6%) to 0.4205 *x* /(1-51.6%) g ⋅ d^-1^. Therefore the final SGR formula should be:

$${SGR}_{min}/\%\cdot {\mathrm{d}}^{-1}=\left[\mathit{\ln}\left(8.41+\frac{0.2523x}{1-51.6\%}\right)-\mathit{\ln}8.41\right]\ast 100\%$$

$${SGR}_{max}/\%\cdot {\mathrm{d}}^{-1}=\left[\mathit{\ln}\left(8.41+\frac{0.4205x}{1-51.6\%}\right)-\mathit{\ln}8.41\right]\ast 100\%$$

where *x* is the biomass synthesis rate simulated with *i*crab4556.

## Results and discussion

### Transcriptome sequencing

Total RNA from five mixed tissues, including gill, muscle, thoracic ganglion, eyestalk, and hepatopancreas, were extracted to obtain the *E. sinensis* transcriptome data. The RNA samples had no genomic contamination, protein and impurity contamination, and no color abnormality. The final quality testing result was level A, which was the highest level for eukaryotic transcriptome RNA. Total RNA was used as the input material for the RNA sample preparations and sequenced with the Illumina HiSeqTM2500. A total of 98,358,250 clean reads were obtained with Q20 at 96.02% and Q30 at 90.6%. The GC content of the clean reads was 51.68%. From these clean reads, 449,372 transcripts (mean length 590 nt) were assembled with Trinity software and then, 246,232 unigenes (the longest transcripts of each gene, mean length 851 nt) were constructed. Finally, 10,108 (4.1%), 29,836 (12.11%), and 67,612 (27.45%) unigenes were matched against NR, NT, and PFAM databases, respectively. Also, 68,664 unigenes (27.88% of total unigenes) were annotated to the GO database, including 50,113 assigned to the biological process category, 55,907 assigned to the molecular function category and 38,402 assigned to the cellular component category. Moreover, 25,157 unigenes were assigned to KO IDs.

### GEM reconstruction of *E. sinensis*

The transcriptome sequencing results of *E. sinensis* comprised 246,232 unigenes, including 25,157 unigenes containing KO ID, and 5,846 nonrepetitive KO ID were obtained. The KEGG database contained information of 10,827 reactions, including 6,037 reactions with KO ID. Using KO ID as a bridge, the reaction information from the KEGG database was matched with unigenes in transcriptome data, and a preliminary reconstructed GEM of *E. sinensis* was obtained, which included 1759 metabolic reactions, 1838 metabolites and 4706 unigenes.

The preliminary reconstructed GEM was artificially refined to improve the quality of the network. Through the balance of reaction equation, 59 unbalanced reactions were revised. According to the KEGG pathway diagram and reactant pair information, combined with the characteristics of reaction and reactant, 129 reactions in the network were supplemented with main reactions. The improvement in the main reaction information was of great significance for the subsequent gap filling step of the network. Further, 142 reactions had missing pathways, and 171 reactions had missing subsystem information in the preliminary reconstructed GEM. According to the information provided by the KEGG database, 119 reactions were supplemented with concrete pathways and subsystems. The other reactions were all classified into “metabolic pathway,”

By integrating and deleting multi-step reactions, general reactions, incomplete reactions and macromolecular reactions, 63 redundant reactions in the network were processed. After the treatment of chirality conformations, 11 new redundant reactions were deleted. In the gap filling step, the gaps were identified and filled in the pathway and global scales. A total of 166 and 52 reactions were added to the network to fill the gaps in the pathway and global scales, respectively. Also, 225 WCCs were found in the network before gap filling, while the number decreased to 142 after gap filling on the pathway scale and 110 after gap filling on the global scale. It indicated that the gap filling step significantly increased the connectivity of the network.

According to the nutritional requirements of *E. sinensis*, 160 transfer reactions were added, including 80 exchange reactions and 80 transport reactions. The network was divided into two cellular compartments: intracellular and extracellular, and the metabolites were also divided into two categories: intracellular metabolites and extracellular metabolites. After compartmentalization, there are three types of reactions were found in the network model: (1) the reactants and products of the reaction were all intracellular metabolites, indicating that the reaction was an intracellular reaction; (2) the reactants and products of the reaction were all extracellular metabolites, indicating that the reaction was an exchange reaction; and (3) both intracellular and extracellular metabolites were involved in the reaction, which indicated that the reaction was a transport reaction. If an exchange or transport reaction is set to be irreversible, it means that the transported substance can not be obtained from the feed. If the crab itself cannot synthesize a particular substance, it will lead to the lack of the substance and may eventually lead to the development of diseases or even death. In the simulation, it might show as the failure of biomass synthesis. For example, if the transport reaction of an essential amino acid was set to be irreversible, the amino acid would not be available, and hence the flux of biomass synthesis reaction would be 0. However, if it was the change of an exchange or transport reaction for a nonessential amino acid, the biomass synthesis would not be affected. Therefore, as the nonessential amino acids do not needed to be imported from the feed, the reversibility of transport and exchange reactions for nonessential amino-acids were all set as irreversible.

The refined GEM contained 2055 reactions and 4665 unigenes. Based on the current data, including RNA-seq and KEGG, this network was still incomplete. Many parts of the network were still disconnected, which showed that the network still contained 110 WCCs after correction. These missing parts were largely due to the lack of understanding of *E. sinensis*. This was also the reason why the model still needed to be modified by adding the biomass equation and a series of simulations. Another reason was the limitations of the data itself. The network was constructed based on a limited pool of tissues, and the metabolic process of some substances did not exist in these tissues.

Most of the reactions in the GEM belong to the basic metabolism. Discontinuous growth is an important feature of *E. sinensis*. *E. sinensis* goes through more than 10 cycles of ecdysis throughout its life, and each ecdysis cycle is accompanied by a sharp increase in body size, weight, and development. Ecdysone is an important substance affecting the ecdysis process of *E. sinensis*. Ecdysone is synthesized and transformed into active 20-hydroxyecdysone in the Y organ. 20-hydroxyecdysone exists in many tissues and has the function of inducing ecdysis. On the other hand, 20-hydroxyecdysone is transformed to 20,26-dihydroxyecdysone catalyzed by 26-hydroxylase, thus loses its activity. The formation and inactivation of 20-hydroxyecdysone regulate the ecdysis and growth of *E. sinensis*. The GEM includes the reaction from 20-hydroxyecdysone to 20,26-dihydroxyecdysone (R08143), which is a characteristic reaction of *E. sinensis* as an animal grows in stages through ecdysis.

### Addition of biomass reaction

The biomass includes about 51.6% water, 10.1% protein, 35% fat, 1% carbohydrate, 1.5% ash, some trace elements and other nutrients. In the nutritional component detection, the content of 20 amino acids, 22 fatty acids, 9 trace elements and 2 saccharides in cells were detected. The unit of each component was converted into mmol ⋅ gDW^-1^, and then the content value was used as its coefficient in the biomass equation. The coefficient maintenance substances such as ATP and NADPH were also added to the biomass reaction. Finally, the biomass reaction was composed of the metabolites from the nutritional component detection and the maintenance substances (Supplementary file 2). The precursor composition of the biomass reaction plays an important role in the necessity simulation experiment. If a biomass precursor is not considered in the biomass reaction, the synthesis reaction and pathway of this substance may not be required for growth. Subsequently, the genes, reactions and substances related to this are not necessary. Therefore, the existence of a metabolite in the biomass reaction may affect the necessity of its related reaction, genes and other metabolites. On the contrary, the coefficient of a biomass precursor is very important for the accuracy of the predicted growth rate prediction. The biomass reaction produces the dry weight cells by adding the contents of each precursor. When one wishes to accurately predict the optimal growth rate, the content of each precursor plays an important role.

### Network evaluation

#### Synthesis of biomass and biomass precursors

Taking biomass reaction as the objective function, the maximum value of the biomass synthesis rate was calculated to evaluate the network, taking the upper and lower limits of 2,055 reactions as constraints with the FBA algorithm. After the first calculation, the maximum flux value of biomass reaction was zero, which indicated that there are some defects in the biomass synthesis route of the network.

To identify the defects, the synthesis of each precursor for biomass synthesis was checked with the GEM, including 20 amino acids, 22 fatty acids, 9 trace elements and 2 saccharides. As all the trace elements and saccharides were obtained from the feed, the lower bound of their exchange reactions was set to be -1 mmol ⋅ gDW^-1^ ⋅ h^-1^ for trace elements and -5 mmol ⋅ gDW^-1^ ⋅ h^-1^ for saccharides. The precursors actually requiring verification of the synthetic ability were amino acids and fatty acids. Of the amino acids, 8 amino acids (alanine, asparagine, aspartic acid, glutamine, glutamic acid, glycine, proline, and serine) could be synthesized using the model, and the other 12 (lysine, arginine, methionine, tryptophan, phenylalanine, leucine, histidine, valine, threonine, isoleucine, cysteine, and tyrosine) could not be synthesized. Of the 12 amino acids that could not be synthesized, 10 were essential amino acids that must be obtained from the feed except cysteine and tyrosine. Therefore, the upstream and downstream metabolic flow of cysteine and tyrosine were further carefully checked to find the defects in their synthesis routes [29]. Actually, tyrosine and cysteine are semi-essential amino acids for most animals, which are also known as conditional amino acids, referring to the amino acid that can be synthesized in cells, but the synthetic amount cannot meet the growth needs. It still needs to be absorbed from the environment. Therefore, the lower limit of the exchange reactions for tyrosine and cysteine was modified to -3 mmol ⋅ gDW^-1^ ⋅ h^-1^. In the metabolic flux checking of cysteine synthesis route, it was found that the flux of the metabolic processes from sulfate to sulfite and then to hydrogen sulfide was 0. To complete these processes, the exchange and transport reactions of sulfate were first added to the network to absorb sulfate from the environment as a sulfur source. Then, the reactions from adenylyl sulfate to sulfite (R08553) and from sulfite to hydrogen sulfide (R0858) were added. With these reactions supplemented, the synthesis pathway of cysteine was complete and the maximum synthesis flux of cysteine was 8 mmol ⋅ gDW^-1^ ⋅ h^-1^. In the re-calculation of the biomass synthesizing rate, its maximum flux was 2.6639 gDW ⋅ h^-1^, which indicated that the GEM could synthesize biomass.

In addition, studies on the necessity of fatty acids in *E. sinensis* are few. At present, linoleic acid, linolenic acid, EPA, DHA and APA are considered as necessary fatty acids [23, 24]. The model cannot synthesize these five fatty acids, which is consistent with the literature. According to the transcriptome sequencing results, the pathway involved in the synthesis of unsaturated fatty acids in the metabolic system of *E. sinensis* is extremely incomplete, and no unsaturated fatty acids can be synthesized in cells. In fact, the feed of *E. sinensis* is mostly compound feed, and hence may contain a variety of fatty acids or their precursors. However, no clear evidence is available on the determination of all kinds and contents of fatty acids in the feed. Therefore, the fatty acids that were required for biomass synthesis but could nbot be synthesized using the model were all set to be imported from the feed.

#### Testing basic properties of model

In the testing of leak metabolites, the model still produced glucose, fructose, H_2_O, PPi and H_3_PO_4_ when it was closed. The exchange reaction of the leak metabolites was set as the objective function separately in the closed model, and the flux distribution of the network was calculated with the FBA to find out the reason for the leakage of these metabolites. The reactions with the nonzero flux were carefully checked, revealing that the leakage of metabolites was caused by the material imbalance in the network. Although the material of each reaction in the network was balanced, the combination of some specific reactions caused the material imbalance of the whole network. The leakage of glucose and fructose was caused by R02110: (C_12_H_20_O_10_)_n_ <=> (C_6_H_10_O_5_)_n_ and R02109: (C_6_H_10_O_5_)_n_ + H_2_O <=> (C_12_H_20_O_10_)_n_ + C_6_H_12_O_6_. These two reactions both involved amylose and starch with an uncertain number of monomers, leading to material imbalance when the “n” represented different numbers in the two reactions. Similarly, the leakage of H_2_O, PPi and H_3_PO_4_ was caused by seven RNA-involved reactions (R00437–R00443) with uncertain phosphate groups , resulting in material imbalance. When these reactions were all deleted by setting their both upper and lower bounds as zero, there was no leakage of exchanged metabolites occurred in the model. However, still, leakage of metabolites was observed when demand reactions for each metabolite in the model were added. This was not difficult to understand for a less well studied species. Still, many uncertain issues were present in the metabolic process of *E. sinensis*. Therefore, it was difficult to guarantee no leakage of metabolites in the network.

**Fig. 2:**
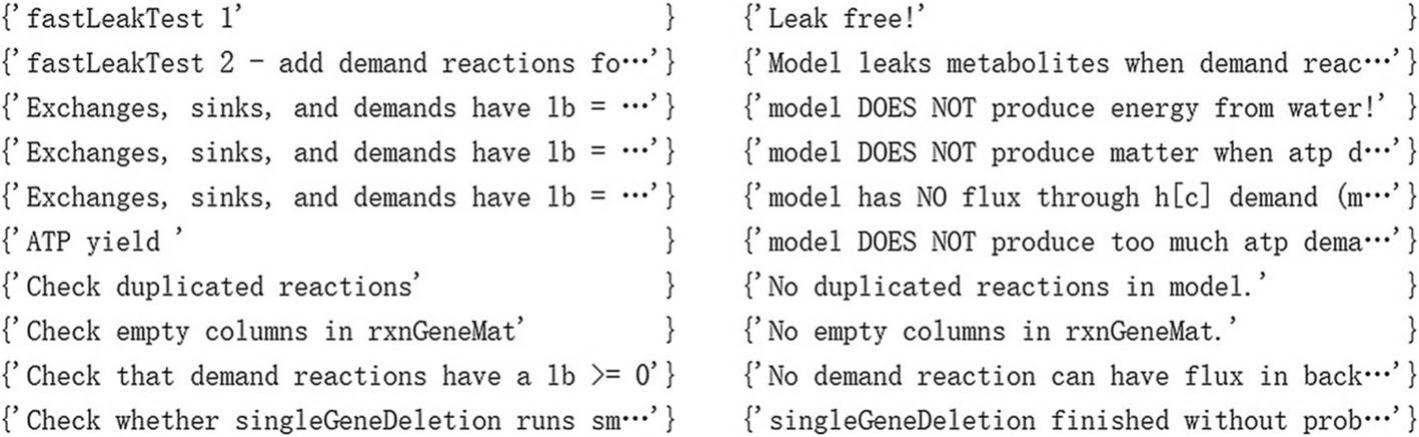
Results of basic properties testing for the model

In the testing of other basic properties tests, the model did not produce energy from water and did not produce matter when ATP demand was reversed. The model had no flux through the h[c] demand and did not produce too much ATP demand from glucose. No duplicated reactions and empty columns were found in the model. No demand reaction could have a flux when demand reactions were in the backward direction. Finally, single-gene deletion ran smoothly. The final testing results are shown in Fig. 2.

**Fig. 3:**
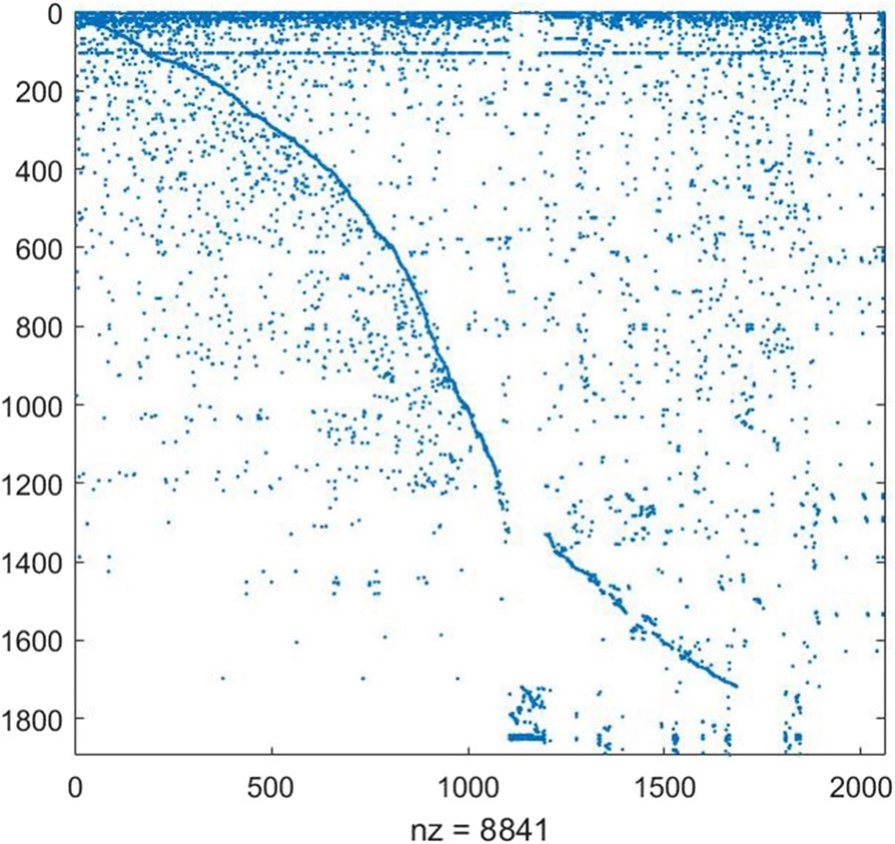
Visualization of stoichiometric matrix. ‘nz’ is the number of nodes in the matrix

The finally reconstructed GEM contained 4665 unigenes, 2060 reactions and 1891 metabolites (Supplementary file 3), named as *i*crab4665. The features of *i*crab4665 are shown in Table 1. The stoichiometric matrix of *i*crab4665 formed by the model transformation with the COBRA toolbox on the MATLAB platform is shown in Fig. 3.

**Table 1:** Features of *i*crab4665

Item	Count
Unigenes	4,665
Reactions	2,060
metabolic reactions	1,897
transport reactions	81
exchange reactions	81
Biomass reaction	1
Metabolites	1,891
cytosol metabolites	1,810
extracellular metabolites	81
Gene-reaction relationship	1,685
Pathways	113
Subsystems	12

### Analysis of the nutritional requirement of *E. sinensis*

Feed plays a key role in crab culture, and the nutritional needs of crabs are the theoretical basis for the design of formulated feed [30, 31]. In this study, the theoretical requirement of nutrients for the accumulating 1 g cell dry weight was calculated based on the GEM of *E. sinensis*. The simulation results were compared with the reported essential amino acids, essential fatty acids and mineral elements to propose constructive suggestions for the feed design.

#### Demand analysis of essential amino acids

The protein requirement of *E. sinensis* can be divided into essential amino acids and nonessential amino acids. *E. sinensis* need 10 kinds of essential amino acids [22]. The lack of essential amino acids leads to malnutrition, molting obstruction, slow growth and development, and even death. Studies have shown that the requirement of essential amino acids is closely related to their composition of essential amino acids in cells. Therefore, some researchers have analyzed the composition of amino acids in *E. sinensis* in different growth stages to estimate the requirement of these amino acids [32–35].

The requirement of essential amino acids was simulated with *i*crab4665 to identify the optimal composition of amino acids in *E. sinensis* feeds. The simulation results were further compared with the literature to check the difference between the theoretical optimal value and the content in current feeds. The unit of the content of amino acids was first transformed into mmol ⋅ gDW^−1^ ⋅ h^−1^ and then compared with the simulation results Table 2.

**Table 2:** Requirements of essential amino acid by *E. sinensis* in literature and simulation results

Essential amino acid	Demand in literature	Demand in mmol⋅gDW^−1^h^−1^	Simulation results	Deposition rate
Arginine	3.62 % [33]	0.2078	0.07	34%
Lysine	2.34% [33]	0.1601	0.0664	41%
Methionine	1.12% [33]	0.0751	0.0249	33%
Leucine	2.36 % [34]	0.1799	0.104	58%
Isoleucine	2.25 % [34]	0.1715	0.0504	29%
Histidine	0.864 % [32]	0.0557	0.0293	53%
Phenylalanine	1.963%.	0.1188	0.0363	31%
Threonine	1.59 % [32]	0.1335	5	-
Tryptophan	0.182 % [32]	0.0089	0.0071	80%
Valine	1.504 % [32]	0.1284	0.0723	56%

The simulation results were lower than the demand for essential amino acids provided in the literature. This might be because the requirement of essential amino acids provided in the literature was the total amount of amino acids to maintain the growth, development, metabolism, and accumulation in the body, while the network simulation results indicated the amount of essential amino acids absorbed by cells to accumulate 1 g of cell dry weight. Some amino acids were preferentially deposited in tissues, while some amino acids might have a more active metabolism, so the amount of essential amino acids deposited in the body was not completely consistent with its requirement. According to the work of Cui et al. [36], the protein deposition rate was calculated as the amount of protein deposition divided by the total protein requirement. Therefore, the deposition rates of the essential amino acids could be obtained by dividing the accumulation requirement of essential amino acids expressed as the simulation results by the total requirement of essential amino acids provided in the literature Table 2, (Supplementary file 4). The deposition rates of arginine, methionine, isoleucine, and phenylalanine were much lower than those of the other amino acids, which indicated that these amino acids might play a more active role in the metabolism of *E. sinensis*. Therefore, the amount of these amino acids in the feed should be proportionally higher than that of other amino acids to fulfill the requirement of *E. sinensis*. In addition, the abnormally large flux of threonine in the simulation results might be due to the lack of key rate-limiting reaction in threonine synthesis or metabolism route in the metabolic network.

#### Demand analysis of essential fatty acids

Crustaceans cannot synthesize polyunsaturated fatty acids from monounsaturated fatty acids. Therefore, it is necessary to provide specific n-3 and n-6 unsaturated fatty acids (UFAs) from the feed to meet their needs for essential fatty acids. The lack of essential fatty acids can inhibit the molting and growth of crustaceans, and even lead to the occurrence of diseases in severe cases. Some crustaceans cannot reproduce smoothly due to the lack of high UFAs [37]. Although several enzymes responsible for the desaturating and elongating of the polyunsaturated fatty acids have been identified in *E. sinensis*, the characterization of these desaturases and elongases has shown that these enzymes are not directly involved in the synthesizing of UFAs [38–40]. Therefore, the demands of UFAs in *E. sinensis* are mainly provided by the diets. Generally speaking, the essential fatty acids of crustaceans are linoleic acid, linolenic acid, EPA and DHA. Therefore, we compared the simulated requirements of these four UFAs to their contents in the literature. The results are shown in Table 3.

**Table 3:** Requirements of essential fatty acids by *E. sinensis* in literature and simulation results

Essential fatty acids	Demand in literature	Demand in mmol⋅gDW^−1^⋅h^−1^	Simulation results
linoleic acid	2.79%	0.0995	0.5835
linolenic acid	0.95%	0.0341	-4.567/0.0024
EPA	0.28%	0.0083	0.069
DHA	0.53%	0.0161	0.0314

The simulation results showed that the theoretical absorption amount of linoleic acid, EPA, and DHA by *E. sinensis* was higher than that reported by Wen et al. Actually, the required amounts of essential fatty acids reported in various examples in the literature are quite different. The types and requirements of lipids in the feed are also variable in different growth stages of *E. sinensis*. Even for the same growth stage, the conclusion about the optimal requirement for the fat content in the feed is not consistent [41]. Wen et al. reported that the optimal requirements of linoleic acid, linolenic acid, EPA, and DHA in the juvenile *E. sinensis* were 2.79%, 0.95%, 0.28%, and 0.53%, respectively [23]. Qi et al. found that the proper requirement of linoleic acid was 1.24% for the juvenile *E. sinensis* with weight 0.22 ± 0.01 g, and the suitable linoleic/linolenic ratio was 0.56-0.74 [42]. Wang et al. [43] showed that when 0.3% of EPA, and DHA were added to the diet, the weight gain and utilization of feed in juvenile crabs were significantly increased. Zhao [44] found that adding appropriate amounts of DHA and DHA/EPA mixture had obvious promoting effects on the growth of juvenile crabs. The suitable DHA content in the juvenile crab feed was about 0.2%, and the suitable DHA/EPA ratio was 2 - 3. Xu et al. [45] found that EPA and DHA had a significant effect on the weight gain of juvenile crabs, and the growth of juvenile crab slowed down when their relative content was lower than 0.03% and 5%, respectively. However, this study was published earlier, and the conclusion that DHA in the feed should not be less than 5% was clearly contradicted in later studies. In addition, Wang et al. [24] reported that arachidonic acid (20:4n-6, ARA) was also an essential for *E. sinensis*. The ARA content in the feed they reported was suitable for adult crabs weighing about 150 - 200 g, and the measurement and prediction in this study were based on juvenile crabs. Therefore, we did not compare the results of the ARA demand with it. In summary, linoleic acid, EPA and DHA were essential fatty acids to promote the growth of juvenile crabs, which was consistent with the simulation results. According to the simulation results, increasing the amount of linoleic acid, EPA, and DHA in the feed should be considered to promote the growth of *E. sinensis*.

The simulation results of linolenic acid were different for various types of linolenic acid. The α-linolenic acid was accumulated at the rate of 4.567 mmol ⋅ gDW^-1^ ⋅ h^-1^ and γ-linolenic acid was absorbed at the rate of 0.0024 mmol ⋅ gDW^-1^ ⋅ h^-1^. The metabolic flux analysis of α-linolenic acid demonstrated that the flux of α-linolenic acid accumulateion R07859 (from lecithin to α-linolenic acid) was 4.606 mmol ⋅ gDW^-1^ ⋅ h^-1^, but with no consumption reaction for α-linolenic acid, which resulted in the final accumulation of α-linolenic acid in the simulation result. In addition, the result of nutritional component analysis also showed α-linolenic acid accumulation in *E. sinensis* cells.

#### Requirement analysis for mineral elements

Mineral elements are indispensable nutrients of *E. sinensis* for maintaining the growth, development, health and reproduction. Unlike terrestrial animals, aquatic animals can absorb minerals from the aqueous environment relying on organs such as the mumps and intestines. Therefore, it is not necessary to supply mineral elements in the feed. However, in the whole life cycle of *E. sinensis*, the environment of its growth and development is mostly fresh water. The inorganic salt content in fresh water is relatively deficient. Therefore, *E. sinensis*, unlike other aquatic animals, tends to require mineral supplementation from the feed to meet its growth needs. The optimal requirements of mineral elements were simulated with *i*crab4665 and compared with the literature Table 4. The demands for calcium and magnesium in the literature were expressed by the content of calcium carbonate and L-aspartate magnesium in the feed. Therefore, the molar amounts of calcium and magnesium were calculated using the molar mass of calcium carbonate and L-aspartate magnesium in unit conversion. The demands for zinc, selenium and copper in literature were expressed as the content of each element in feed, so the molar amounts were calculated using the molar mass of these elements.

**Table 4:** Requirements of mineral elements by *E. sinensis* in literature and simulation

Mineral elements	Mineral elements source	Demand in literature	Demand in mmol⋅gDW^−1^⋅h^−1^	Simulation results
Calcium	Carbonate	2.9 % [46]	0.2897	0.0437
Magnesium	L-aspartate	3.76g/kg [47]	0.0239	0.0233
Zinc	Methionine zinc	20mg/kg [48]	0.0003	0.0005
Selenium	Yeast selenium	0.59mg/kg [49]	0.000007	0.000015
Copper	Sulphate	24.66mg/kg [50]	0.0004	0.0003

The requirements of magnesium, zinc, selenium and copper in the literature were quite close to the simulation results, which reflected the high quality of *i*crab4665. According to the simulation results, the feed addition of zinc and selenium was slightly increased. The requirement of calcium is complex for crabs, because that the calcium content in water and the different growth stages of crabs will all influence the calcium requirements in feed. For example, crabs in molting stage need large amounts of calcium to satisfy the renewal of shell and body growth. Therefore, the demand for dietary calcium for *E. sinensis* should be considered in combination with many factors, which may be the reason for the big difference in the calcium requirement between the literature and the simulation result.

#### Evaluation of the growth rate simulated with *i*crab4665

In order to further evaluate the growth rate of *E. sinensis* simulated with *i*crab4665, we set the maximum absorption rate of nutrients according to the values in the feed in the literature mentioned above (with 1g feed absorbtion per day) and simulated the maximum synthesis rate of biomass. The absorption rate of substances not mentioned in the literature remained unchanged. The final biomass synthesis rate was 0.1203 gDW ⋅ d^-1^. The final SGR for simulation results was 0.75% - 1.24%. Hu et al. investigated the growth rate of juvenile crabs in the indoor tank for three different families and found that their SGR varied from 0.997% - 1.454% [28], which was in good agreement with our simulation results. The small differences between simulation results and the literature might be due to the differences in culture conditions, temperature or water quality.

In the intensive culture mode, a large amount of nitrogen, phosphorus, and other nutrients in the feed with an unreasonable high nutrition ratio are directly discharged into the water without being absorbed by *E. sinensis*, which is an important component of water pollution [51]. In the evaluation of growth rate, although we set the maximum absorption rate of nutrients according to the literature, not all nutrients could be absorbed because the feed composition in the literature was not optimal. The calculated nutrient absorptions of many nutrients were much less than their content in the feed (Supplementary file 5). The unabsorbed part will re-entered the water with the excreta of *E. sinensis*, resulting in the enrichment of nutrients such as nitrogen and phosphorus in water to cause water pollution. Therefore, an important way to reduce water pollution is to optimize the nutrient content in the feed, make full use of the nutrition in the feed and reduce the excretion of excess nutrients.

To further determine the key genes, reactions and pathways that play a role in growth, the flux comparison method can be used [52]. Firstly, the biomass synthesis rate were set to be a larger value and a smaller value, for example, 2.6636 gDW ⋅ h^-1^ (the maximum value) and 1.0 gDW ⋅ h^-1^, and the flux distribution in the GEM were calculated, respectively. Then, the flux of each reaction in the two calculations was compared to determine the key reactions and pathways. After comparison, the flux of 194 reactions were found changed when the biomass synthesize rate changes. Except the transport and exchange reactions, the flux-changed reactions were mostly distributed in carbohydrate metabolism (37 reactions), nucleic acid metabolism (13 reactions) and amino acid metabolism (20 reactions). The top 10 reactions with largest flux changes were distributed in galactose metabolism, pyruvate metabolism, pyrimidine metabolism, synthesis and degradation of ketone bodies, and several amino acid metabolism pathways. The flux-changed reactions and their corresponding unigenes, pathways and subsystems were listed in Supplementary file 6.

## Conclusion

Optimization of feed is crucial for cultivating aquatic products, and traditional methods usually pass trial and error to pick out suitable additions. In this study, we reconstructed the largest GEM of *E. sinensis i*crab4665. The GEM was used to conduct the *in silico* prediction on the optimal proportion of feed suitable for the growth of *E. sinensis* and put forward reasonable suggestions for optimizing of feed. Based on the existing data, the simulation results of the model were not completely consistent with the experimental results. This difference was not only the reason for the completeness of the model, but also affected by the experimental results. For example, the literature values for amino acids might also be influenced by the experimental conditions or other factors, and this limited the analysis of the deposition rate. Further experiments, for example, isotope labeling experiments, may need to be applied to clarify the exact amount of metabolized and deposited amino acids. It can also be helpful for the study of the metabolic flow of amino acids. Referring to the metabolic process of amino acids in other animals, the active metabolized amino acids in a cell may be transported to other tissues with the blood to synthesize proteins with specific functions as needed. They may also be used to synthesize proteins in the hepatopancreas for storage, or become an important component of cells, so as to make *E. sinensis* more nutritious. It would be more accurate to analyze the metabolic flow of amino acids through the combination of *in silico* prediction and *in vivo* experiments.

In addition, the prediction results also need to be further verified by feeding experiments. The impact of nutrients proportion on the cost of the feed is not easy to be estimated. Based on the simulated optimal nutrients proportion, the proportion of some nutrients needs to be increased, while relatively, the proportion of other nutrients will be reduced. Therefore, the final change of feed cost should be determined according to the market value of these nutrients. However, a reasonable feed ratio is beneficial to the growth of E. sinensis. Therefore, even if the cost of the feed remains unchanged or even increases after optimization, considering that the value of adult crab is much higher than that of feed, it is likely to be cost-effective in terms of cost performance. Besides, since the cost of pure nutrients is relatively high, from the perspective of cost, a feed can be prepared with specific common feed components according to the nutritional needs simulated by the model, so as to reduce the feeding cost of *E. sinensis*. The feeding experiments should be carried out in follow-up studies to verify the accuracy of the model prediction, and provide new data for further improvement of the model.

One important thing to be noted is that the growth of *E. sinensis* belongs to metamorphosis development, therefore, the difference in metabolic processes between its different growth stages is extremely large. Therefore, the life stage of *E. sinensis* used in generating transcriptome data will affects the GEM a lot. The growth of *E.sinensis* includes five stages: fertilized egg, daphnia larva, megalopa larva, juvenile and adult, in which juvenile is the longest stage with the fastest growth rate as well. In the process of *E.sinensis* breeding, the growth of juvenile stage directly affects the size and quality of the adult crabs. Therefore, the *E.sinensis* in juvenile stage was chosen to reconstruct the GEM in this study and the GEM was only applicable to the feed formulation of *E. sinensis* for the juvenile stage. On the conotrary, the limited pool of tissues may also influence the comparison of simulated amino acids, fatty acids, and mineral composition with the data from the literature, as these are very lifecycle-dependent. During the juvenile stage, the crab maintains the same shape and grows through molting until adulthood. Therefore, the size of the juvenile crab used for RNA-seq has no major effect on the GEM.

Although the model still has many deficiencies to be improved, this study promoted the exploration of feed optimization for aquatic crustaceans from *in vivo* to *in silico*. The results might contribute to the improving feed efficiency and be beneficial for improving the aquaculture production of *E. sinensis*.

## Supplementary Information

**Additional file 1:  Supplementary file 1.** Detail procedure of transcriptome sequencing and refinement of the GEM.

**Additional file 2: Supplementary file 2. **The results of nutritional composition detection of *E. sinensis.*

**Additional file 3: Supplementary file 3. **The reconstructed model *i*crab4665.

**Additional file 4: Supplementary file 4.** The calculation process of Table 2.

**Additional file 5: Supplementary file 5.** The nutrient requirement in literature and simulation results when setting limits according to literature.

**Additional file 6: Supplementary file 6.** The list of reactions with flux changed when the biomass synthesis rate changes.

## Data Availability

The RNA-seq data is availability from the Gene Expression Omnibus (GEO) database wih the accession number GSE182818. The GEM and nutrient requirements analysis results are availability from the supplementary files.

## Abbreviations

GEM: genome-scale metabolic network model
PCR: polymerase chain reaction
GEO: Gene Expression Omnibus
VBA: Visual Basic for Applications
WCCs: weakly connected components
FBA: Flux balance analysis
SGR: specific growth rate
UFAs: unsaturated fatty acids
