# Supplementary material for: Genome-scale metabolic network model of Eriocheir sinensis icrab4665 and nutritional requirement analysis
Source: BMC Genomics. 2022 Jun 28;23:475. doi: 10.1186/s12864-022-08698-z (PMC9238104; doi:10.1186/s12864-022-08698-z)
Supplement: Supplementary file 1 — Additional file 1: Supplementary file 1. Detail procedure of transcriptome sequencing and refinement of the GEM. [file 12864_2022_8698_MOESM1_ESM.docx]

### The transcriptome sequencing procedure

The total RNA was extracted using the TRIzol method (Invitrogen) for transcriptome analysis. The quantity and quality of total RNA were assessed using an RNA Nano 6000 Assay Kit of a Bioanalyzer 2100 system (Agilent Technologies, CA, USA). The concentration, integrity, and purity of the extracted RNA were checked. Sequencing libraries were generated using an NEBNext® Ultra™ RNA Library Prep Kit for Illumina® (NEB, USA) following the manufacturer's recommendations. Briefly, mRNA was purified from total RNA using poly-T oligo-attached magnetic beads. Fragmentation was carried out using divalent cations at an elevated temperature in NEBNext First Strand Synthesis Reaction Buffer (5×). The first-strand cDNA was synthesized using random hexamer primer and M-MuLV Reverse Transcriptase (RNase H-). The second-strand cDNA synthesis was subsequently synthesized using DNA Polymerase I and RNase H. The remaining overhangs were converted into blunt ends via exonuclease/polymerase activities. After adenylation of 3’ ends of DNA fragments, the NEBNext Adaptor with a hairpin loop structure was ligated to prepare for hybridization. The library fragments were purified with an AMPure XP system (Beckman Coulter, Beverly, USA) to select cDNA fragments of preferentially 150–200 bp in length. Then, 3 µL of the USER Enzyme (NEB, USA) was used with size-selected, adaptor-ligated cDNA at 37°C for 15 min followed by 5 min at 95 °C before polymerase chain reaction (PCR). Subsequenty, PCR was performed with Phusion High-Fidelity DNA polymerase, universal PCR primers and index (X) primer. At last, PCR products were purified (AMPure XP system), and library quality was assessed on the Agilent Bioanalyzer 2100 system. The clustering of the index-coded samples was performed on a cBot Cluster Generation System using a TruSeq PE Cluster Kit v3-cBot-HS (Illumina) following the manufacturer's protocols. After cluster generation, the library preparations were sequenced on an Illumina Hiseq 2500 platform.

The original image data were transferred into sequencing data by base calling, which were defined as raw reads. These reads were processed through in-house Perl scripts. In this step, clean data (clean reads) were obtained by removing reads containing an adapter, reads containing poly-N and low quality reads from raw data. At the same time, Q20, Q30, and GC contents of the clean data were calculated. All the downstream analyses were based on the clean data with high quality. Then, the transcriptome de novo assembly was carried out with the short-reads assembling program-Trinity to generate transcripts. The longest transcript generated with Trinity for a gene was identified as a unigene. The sequenced unigenes were subsequently aligned against the NR and Nt databases using BLAST searching with an E-value < 1*E-5 and the PFAM database using the HUMMER package with an E-value < 0.01. The GO annotation of unigenes was obtained using the Blast2GO program based on NR and PFAM annotations with an E-value cutoff at 1*E−6. The KO and pathway annotations were obtained by KAAS (KEGG Automatic Annotation Server) with an E-value cutoff at 1*E−10.

### Refinement procedure of the GEM

### Balancing of the reaction equation

Based on the principle of element conservation, we compared the number of elements on the left- and right- hand sides of each reaction equation. A balanced reaction is one with conservation of elements (whose types and number of elements on the left and right sides are equal), while an unbalanced reaction is an opposite. The unbalanced reaction equations in the model were manually balanced with the principle of element and charge conservation. According to the type and number of missing elements in the reaction, the reaction was balanced with the reaction characteristic information. The element and charge balance was checked with the program wrote with VBA language, and the substances added for reaction balance were determined manually. A vast majority of unbalanced reactions were caused by the lack of hydrogen or oxygen. For these reactions, hydrogen ions, electrons, water molecules, oxygen or other substances were added accordingly. For example, R10948 (ATP + HCO3- <=> ADP + carboxyphosphate) from the KEGG database had 17 and 18 H elements on the left and right sides of the equation, respectively. Besides, there is one more negative charge was present on the left side than on the right side. Therefore, a hydrogen ion was added to the left side of the equation to maintain the element and charge balance.

### Supplementation of the main reaction, pathway, subsystem and other information

The main reaction is the reaction composed of the main substances participating in the reaction. Generally, the currency metabolites such as ATP, ADP, NADH, NAD^+^, H_2_O, NADPH, NADP^+^ and H^+^ are removed from the reaction equation to obtain the main reaction. The main reactions reflect the overall structure of the network and are important to the following gap filling step. The main reactions for some reactions were missing in the download files from KEGG database. The missing main reactions were added according to the KEGG pathway map. If the main participants of a reaction were not clear in the pathway map, the appropriate reactant pairs provided in the RCALSS information of the KEGG database were referenced to determine the main participants in the reaction. The RCLASS consists of reaction class entries defined by the identity of chemical transformation patterns in the “main” reactant pairs that generally correspond to the main substrate and product pairs shown on the KEGG metabolic pathway maps. Each RCLASS entry indicates a collection of substrate–product pairs that appear on the KEGG metabolic pathway maps and whose chemical transformation patterns are identical [[1](#_ENREF_1), [2](#_ENREF_2)].

For the reactions without pathways, the pathway information was determined according to the following principles: (1) if the substrates products of a reaction belonged to the same pathway, then, this pathway was selected preferentially; (2) the pathway containing the key reactant in a reaction was determined as the pathway of this reaction; (3) if the pathway could not be determined according to steps (1) and (2), the reaction was classified to “metabolic pathway.” After the pathway information was determined, the subsystem information was supplemented according to the pathway-subsystem relationship downloaded from the KEGG database.

### Deletion of redundant reactions

Redundant reactions such as step reaction, general reaction, incomplete reaction, and macromolecular reaction need to be further processed to simplify the network and ensure the reasonable distribution of metabolic flux. In a GEM, the coexistence of total reaction and step reactions leads to the disorder of metabolic flux distribution. Therefore, it is necessary to make a choice between them. If the metabolites participating in the step reactions were not involved in other reactions in the network, the total reaction was retained. Otherwise, the step reactions were retained.

The KEGG database annotates some reactions that can be divided into the same category as a general reaction. For example, reaction R00056 (dinucleotide + H_2_O <=> 2 mononucleotide) refers to the hydrolysis of a dinucleotide to a single nucleotide, but the exact nucleic acid type is not determined. The metabolites in general reactions are unclear and will influence the simulation results. Therefore, the general reactions were deleted from the network.

Incomplete reaction means that some substances involved in the reaction have not been confirmed by experiments and added to the reaction equation. Incomplete reactions disturb the conservation of elements. Therefore, they were removed from the network.

In KEGG database, the coefficients of many macromolecular reactions are represented by unknown numbers like m and n. Such reactions will lead to incalculable flux distribution of the network, so they were deleted from the network.

The deletion of general reactions, incomplete reactions and macromolecular reactions may have some effect on the growth calculation. However, because these reactions are incomplete or unclear, retaining them will also mislead the calculation of the model and even lead to the incalculability of the model. Therefore, these reactions were removed in the process of network refinement.

### Standardization of metabolites

In the KEGG database, metabolites with different chirality are assigned to different IDs. Taking glucose as an example, glucose has three IDs in the KEGG database, which are C00031 (D-glucose), C00267 (alpha-D-glucose), and C00221 (beta-D-glucose). Actually, there are only alpha-D-glucose and beta-D-glucose in cells. In order to prevent the confusion of recognition caused by unclear chirality in the network, D-glucose in the reaction equations was all changed to alpha-D-glucose, which is more likely to appear in animal cells [[3](#_ENREF_3)]. At the same time, the redundant reactions due to the standardization of metabolites were removed. Finally, the IDs of D-fructose, D-galactose, D-glucose-6-phosphate, D-fructose-6-phosphate and D-fructose-1,6-phosphate were all standardized to their alpha chirality.

### Gap filling

The refined network is not fully connected because of the incomplete annotation and understanding of the cellular metabolic system. The gaps in the network will affect the network connectivity and accuracy of the simulation. To fill the gaps as much as possible, each pathway of the network was first divided into different weakly connected components (WCCs) to find the breakpoints in the pathway scale [[4](#_ENREF_4)]. Then, the reactions that could connect two different WCCs were searched in the range of all the reactions in the KEGG database. All the 10,827 reactions in the KEGG database were used for the gap filling. Each reaction contained in the KEGG database but not in the GEM was checked whether it can be used as a gap-filling reaction. The reactions found were added to the pathway to fill the gaps. When a gap had multiple candidate gap-filling reactions, the reaction located in the same pathway with the WCCs it connected was preferred. Gap filling is an iterative step. After each turn of gap-filling, all the reactions not included in the GEM was checked again weather there was any reaction that can be filled into the network due to last round of gap filling. The gap filling step iterated until no reaction can be found to fill into the GEM. In addition, in order to truly reconstruct the actual biological pathway, the gaps were filled both on the pathway and global scales. These two steps were run sequentially to fill the gaps in the network to a maximum extent [[5](#_ENREF_5)]. Gap filling on the pathway scale prefers to increase the connectivity in each pathway, whereas that on the global scale inclines to increase the connectivity of the whole network.

### Addition of transfer reactions

The growth of organisms is inseparable from the material exchange with the environment, including the absorption and utilization of nutrients, and the discharge of metabolic wastes. Transfer reactions were added to the GEM according to the characteristics of species to fit the actual situation of biological metabolism and simulate the metabolic process more accurately. Transfer reactions include transport reactions and exchange reactions. Transport reaction is used to transport metabolites between cytosol and extracellular compartment, which is expressed as follows: intracellular metabolite <=> extracellular metabolite. Exchange reaction refers to the processes involving the absorption of the nutrients from the environment or discharge of the metabolic wastes, which is expressed as: extracellular metabolite <=>.

### Addition of cellular compartments

The reactions in GEM were divided into two compartments: cytosol and extracellular. All metabolites were also divided into two types: cytosol metabolites (expressed by [c]) and extracellular metabolites (expressed by [e]).

1. Muto A, Kotera M, Tokimatsu T, Nakagawa Z, Goto S, Kanehisa M: **Modular architecture of metabolic pathways revealed by conserved sequences of reactions**. *Journal of chemical information and modeling* 2013, **53**(3):613-622.

2. Kanehisa M, Sato Y, Kawashima M, Furumichi M, Tanabe M: **KEGG as a reference resource for gene and protein annotation**. *Nucleic acids research* 2016, **44**(D1):D457-462.

3. Mykles DL: **Ecdysteroid metabolism in crustaceans**. *The Journal of steroid biochemistry and molecular biology* 2011, **127**(3-5):196-203.

4. Hao T, Ma HW, Zhao XM, Goryanin I: **Compartmentalization of the Edinburgh Human Metabolic Network**. *Bmc Bioinformatics* 2010, **11**.

5. Hao T, Ma HW, Zhao XM, Goryanin I: **The reconstruction and analysis of tissue specific human metabolic networks**. *Molecular Biosystems* 2012, **8**(2):663-670.
