# Supplementary material for: Genome-scale metabolic network model of Eriocheir sinensis icrab4665 and nutritional requirement analysis
Source: BMC Genomics. 2022 Jun 28;23:475. doi: 10.1186/s12864-022-08698-z (PMC9238104; doi:10.1186/s12864-022-08698-z)
Supplement: Supplementary file 4 — Additional file 4: Supplementary file 4. The calculation process of Table 2. [file 12864_2022_8698_MOESM4_ESM.docx]

**The Calculation process of Table 2**

Taking arginine as an example，

1. It is suggested in the literature [[1](#_ENREF_1)] that the demand for arginine of juvenile *E. sinensis* is 3.62%, then 1 g feed contains 0.0362 g arginine. The molar mass of arginine is 174.201 g / mol, so the molar content of arginine in feed is 0.0362/174.201 × 1000 = 0.2078 mmol / g.
2. In the simulation, the biomass synthesis rate was set to be 1.0 gDW⋅h^-1^. Assuming that all the feed can be absorbed and used for growth, if the biomass synthesis rate is 1.0 gDW per hour, the feed requirement rate is 1.0 g/gDW^−1^h^−1^. Therefore, the requirement of arginine is 0.2078 × 1.0 = 0.2078 mmol⋅gDW^−1^h^−1^.
3. According to the work of Cui et al. [[2](#_ENREF_2)], the protein deposition rate was calculated as the amount of protein deposition divided by the total protein requirement. Therefore, the deposition rates of the essential amino acids was obtained by dividing the accumulation requirement of essential amino acids expressed as the simulation results by the total requirement of essential amino acids provided in the literature.

**Reference**

1. Ye JY, Wang HY, Guo JL, Chen JM, Pan X, Shen BQ: **Lysine,methionine and arginine requirements of junvenile Chinese mitten crab (Eriocheir sinensis)**. *JOURNAL OF FISHERIES OF CHINA* 2010, **34**(10):1541-1548.

2. Cui YY, Zhang NN, MA QQ, Chen Q, Shen ZH, Du ZY, Chen LQ: **Effects of four commonly used plant protein sources on growth performance,amino acids retention and antioxidant enzyme activities in juvenile Chinese mitten crab,Eriochier sinensis**. *Acta hydrobiologica sinica* 2017, **41**(01):146-154.
